# A Longitudinal Study of the Feline Faecal Microbiome Identifies Changes into Early Adulthood Irrespective of Sexual Development

**DOI:** 10.1371/journal.pone.0144881

**Published:** 2015-12-14

**Authors:** Oliver Deusch, Ciaran O’Flynn, Alison Colyer, Kelly S. Swanson, David Allaway, Penelope Morris

**Affiliations:** 1 WALTHAM Centre for Pet Nutrition, Freeby Lane, Waltham-on-the-Wolds, Leicestershire, United Kingdom; 2 Department of Animal Sciences, University of Illinois, Urbana, Illinois, United States of America; 3 Division of Nutritional Sciences, University of Illinois, Urbana, Illinois, United States of America; 4 Department of Veterinary Clinical Medicine, University of Illinois, Urbana, Illinois, United States of America; National Cancer Institute, UNITED STATES

## Abstract

Companion animals provide an excellent model for studies of the gut microbiome because potential confounders such as diet and environment can be more readily controlled for than in humans. Additionally, domestic cats and dogs are typically neutered early in life, enabling an investigation into the potential effect of sex hormones on the microbiome. In a longitudinal study to investigate the potential effects of neutering, neutering age and gender on the gut microbiome during growth, the faeces of kittens (16 male, 14 female) were sampled at 18, 30 and 42 weeks of age. DNA was shotgun sequenced on the Illumina platform and sequence reads were annotated for taxonomy and function by comparison to a database of protein coding genes. In a statistical analysis of diversity, taxonomy and functional potential of the microbiomes, age was identified as the only factor with significant associations. No significant effects were detected for gender, neutering, or age when neutered (19 or 31 weeks). At 18 weeks of age the microbiome was dominated by the genera *Lactobacillus* and *Bifidobacterium* (35% and 20% average abundance). Structural and functional diversity was significantly increased by week 30 but there was no further significant increase. At 42 weeks of age the most abundant genera were *Bacteroides* (16%), *Prevotella* (14%) and *Megasphaera* (8%). Significant differences in functional potential included an enrichment for genes in energy metabolism (carbon metabolism and oxidative phosphorylation) and depletion in cell motility (flagella and chemotaxis). We conclude that the feline faecal microbiome is predominantly determined by age when diet and environment are controlled for. We suggest this finding may also be informative for studies of the human microbiome, where control over such factors is usually limited.

## Introduction

In the last decade, advances in DNA sequencing technologies have accelerated the culture-independent study of microbial communities and improved our understanding of the microbiome (the combined genetic material of a community and the functional potential of its protein coding genes). One area of particular interest is the human microbiome and its relevance to health, disease and nutrition. The finding that the microbiome is often a better predictor of complex metabolic diseases (such as diabetes, cardiovascular disease and obesity) than genetic markers [1,2] underpins its importance to health. A wide variety of factors has been shown to be associated with the gut microbiomes of healthy individuals including diet [3], age [4] and gender [5]. In addition associations with a wide range of diseases such as obesity [6], type 1 diabetes [7] and even autism [8] have been reported. With few exceptions [9,10], studies in humans are generally observational. Intervention studies are typically carried out in rodent models and have demonstrated causal relationships between diet, the microbiome and obesity [11].

The microbiome of companion animals is of interest for a number of reasons. Owners share their home with their pets and an exchange of microbes between pets and owners has been demonstrated [12]. Furthermore, companion animals can suffer from a range of medical conditions such as obesity, cardiovascular disease, diabetes and periodontal disease, for which microbial associations have been demonstrated in humans. Microbiome research in companion animals to date includes but is not limited to the skin of allergic dogs [13], the gingiva of dogs with periodontal disease [14], the faeces of dogs with diarrhoea and inflammatory bowel disease [15] as well as the effect of dietary macronutrients [16,17] and fibre [18] on the faecal microbiome in cats.

Companion animals may be more suitable for studying some aspects of the microbiome than humans. Complete and balanced diets may be fed as the sole source of food for extended periods of time, reducing confounding effects of different dietary preferences between individuals. Longitudinal studies in which the same individuals are tracked over various developmental stages are also more feasible in companion animals. Tracking the same individuals over time may be key in studying certain aspects of the microbiome as observed inter-individual variability may be high [19–21]. While this is also practical in rodent models, cats are of particular interest because they are obligate carnivores and the microbiome of carnivores is not well studied as of today.

Domestic cats also provide an excellent opportunity for studying the impact of hormones on the microbiome. Neutering is a common practice to control reproduction and prevent diseases. Recent surveys reported that >90% of cats in the UK and ~80% of the cats in the US were neutered [22,23]. Traditionally, cats are neutered around six to seven months of age [24] but early neutering (before or at four months of age) is now commonly carried out in the US. Neutering stops the production of sex hormones and allows evaluation of their impact by comparison of the microbiomes of animals of similar age with and without sex hormones. Furthermore, an acute increase in food intake and weight gain following neutering has been reported in cats [25,26], and there is an association between neutering and obesity [27]. Furthermore, injection of estradiol is able to reduce feed intake in neutered cats [28], indicating a role of sex hormones and sexual development in intake.

When cats are neutered there is a risk of acute weight gain, that appears to be due to changes in both feeding behaviour (caloric intake and macronutrient selection) and metabolism (13–37% reduction in energy requirements post-neuter required to maintain the pre-neuter, stable body weight in adult cats[25–27]. To maintain adult cats at their ideal body weight post-neuter, it has been reported that caloric intake needs to be reduced by 25–30%. This suggests that cats either reduce the energy expenditure or increase their energy extraction from the diet, for example by increasing the fermentative bacterial population that produce short-chain fatty acids that can be readily absorbed by the host.

In this study a cohort of 30 kittens (16 males and 14 females) that were neutered at either 19 weeks (early) or at 31 weeks (conventional) and fed a single batch of dry diet to maintain an ideal body condition score through growth, had their faecal microbiome characterised (as surrogate for the gut) at 18, 30 and 42 weeks of age. By controlling for diet and food intake, investigations into potential associations with age, gender, neutering and age when neutered were possible. This also provides an opportunity to determine whether a hormone-led change in microbiome could influence this acute post-neuter weight gain.

## Results

DNA obtained from 88 fresh faecal samples collected from 30 kittens that had previously been assigned to either an early or conventional neuter group (19 or 31 weeks of age) and sampled at 18, 30 and 42 weeks of age was sequenced on the Illumina Hi-Seq for metagenome analysis. A total of 5.0 billion shotgun reads of 100 bases in length were generated (501 Gb) which resulted in 4.8 billion quality reads after trimming (approx. 479 Gb) with an average of 55 million reads per sample (range: 23 to 91 million reads). A composition vector based binning approach (MetaCV [29]) against a reference database of eleven million protein sequences from 2,059 fully-sequenced genomes (2,049 bacteria, 4 archaea and 6 viruses) yielded average annotation rates of 35 and 24 percent for taxonomy (genus) and ortholog gene groups (KEGG level 4), respectively (S1 Table). The average annotation rate was higher at 18 weeks of age (42% and 28% for genus and KEGG level 4, respectively) than at 30 (32% and 22%) and 42 weeks of age (31% and 22%).

**Fig 1:**
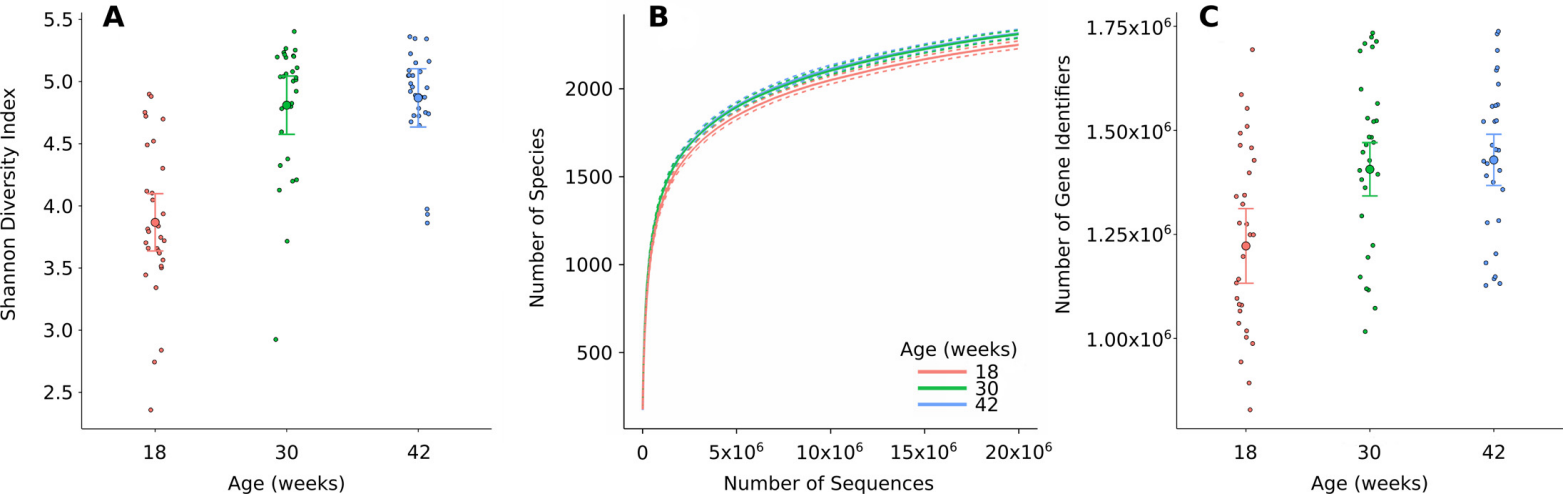
Species diversity and richness at 18, 30 and 42 weeks of age. (a) Shannon diversity indices as inferred from normalized species counts. (b) Rarefaction analysis of species richness. (c) Gene richness shown as the number of unique protein coding genes (unique GI numbers). Species diversity, species richness and gene richness increased with age but only differences from week 18 to weeks 30 and 42 were statistically significant. No significant associations with neuter date or gender were found (S3 and S4 Tables).

In total, 33 phyla, 238 families, 605 genera and 1,113 species of prokaryotes were identified in feline faeces (S2 Table). Each level of the taxonomic hierarchy was dominated by just a few taxa (e.g. the 53 most abundant genera had cumulative sequence counts of 80%) and contained many rare taxa (e.g. the 95 least abundant genera had cumulative sequence counts of ≤ 1%). Shannon diversity indices ranged from 2.4 to 5.4 with an average of 4.5 and were analysed by linear mixed models. Diversity increased from week 18 (3.9) to week 30 (4.8) and 42 (4.9). Increases in diversity from week 18 to 30 and from week 18 to 42 were significant at *p*<10^−5^ (Fig 1A and S3 Table). Rarefaction analysis showed a sharp rise in the number of species with increasing sequence depth (Fig 1B). At 5 million sequences, which correspond to less than ten percent of the average number of sequences per sample, approximately 80% of the taxa were observed indicating deep sequencing. A statistical analysis of rarefaction data detected small but significant differences between ages at *p* = 0.001 (S4 Table). No significant associations were found with gender, neutering or age when neutered at *p*<0.05.

**Fig 2:**
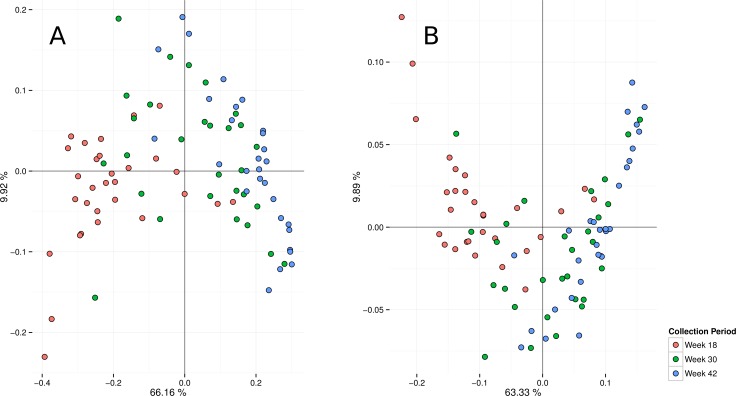
Principal coordinates analysis (PCoA) of microbiome taxonomy and function. Analyses of genus (a) and KEGG ortholog group data (b) indicated a structure based on age (weeks).

A Principal Coordinate Analysis (PCoA) of relative genus abundance data indicated a structure mostly consistent with age (Fig 2A). The majority of the variance (66%) was explained by the first component plotted on the x-axis with mostly samples from week 18 on the left, and those from week 42 on the right. Week 30 samples were more dispersed and PCoA ordinations on the higher levels of taxonomy indicated a similar structure (S1 Fig). Individual samples from the same week differed considerably in the composition of the most abundant genera indicating a high inter-individual variability (S2 Fig). A hierarchical clustering approach indicated a similar structure to the genus PCoA (Fig 2A) although with a less well resolved structure (especially for week 30 samples).

**Fig 3:**
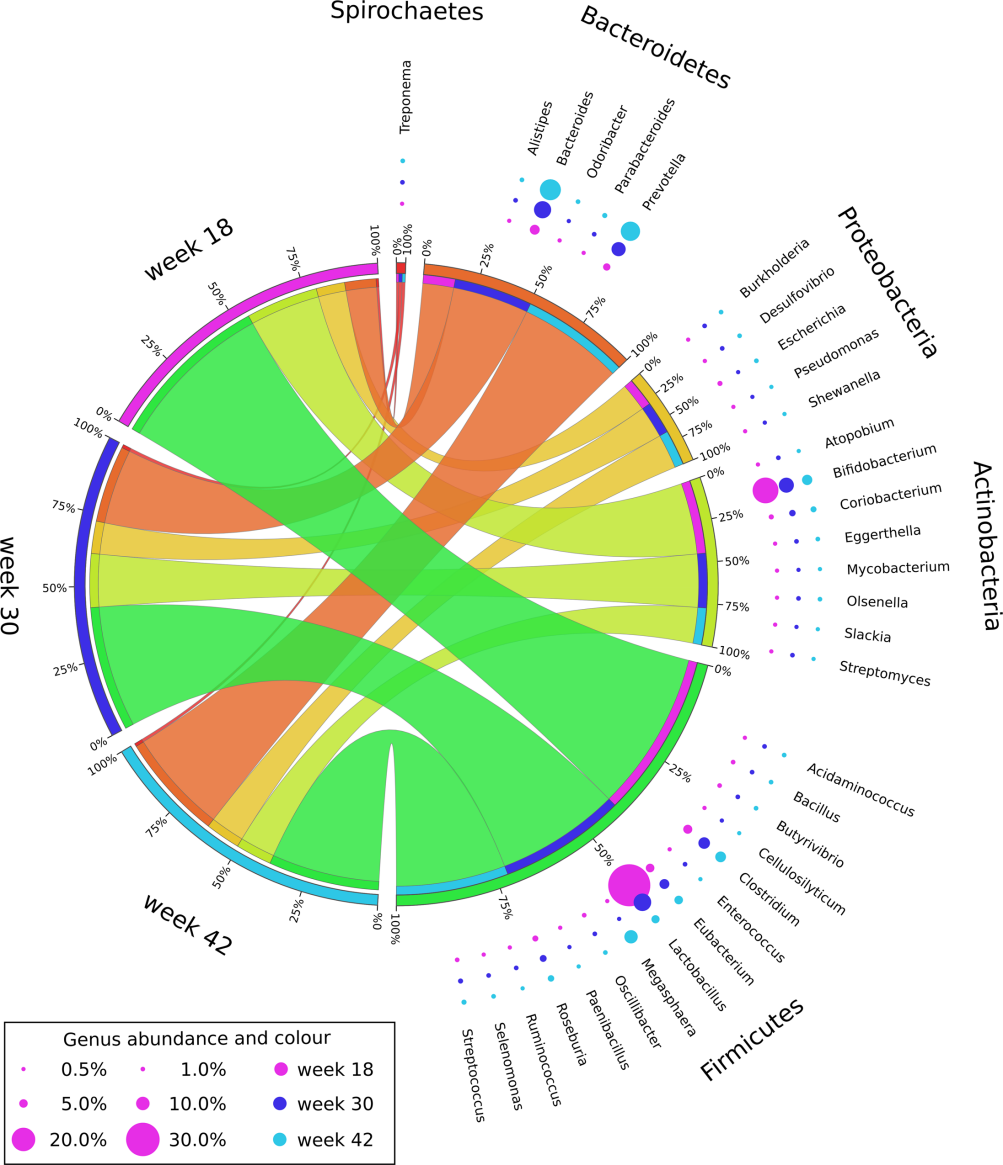
Genus and phylum composition of the feline faecal microbiome. The peripheral bubble plot illustrates the average relative abundance of the 34 most abundant genera (≥ 0.3% average relative abundance at ≥ 1 time point) at 18, 30 and 42 weeks of age (from inside to outside). The central circular plot illustrates the community structure at the phylum level (five most abundant phyla shown) allowing for quick inference of phylum level composition at a given age as well as temporal distribution of a phylum. For example: at week 18 the microbiome is composed of 53% Firmicutes, 23% Actinobacteria, 9% Proteobacteria, 10% Bacteroidetes and 1% Spirochaetes (scaled to total 100% in plot); the phylum Firmicutes decreases from 53% at week 18 to 41% at week 30 and 36% at week 42. Of note are decreases in *Bifidobacterium* and *Lactobacillus* and increases of *Bacteroides* and *Prevotella* over time. A tabular representation of the underlying data is given in Table 2.

Relative abundance data were analysed statistically by generalized estimating equations for associations with age, gender and neuter age (19 or 31 weeks) (see Table 1 for a summary). Statistically significant associations with age were identified for 589 out of 605 genera and 233 out of 238 families (with *p*<0.00008 and 0.00022 respectively using a Sidak adjustment to maintain an overall error rate of 5%, S6 Table). Odds increased with age for 585 genera; 4 genera had decreased odds. No significant associations with gender and neuter age were found at the given *p*-value thresholds. The most abundant phyla and genera and their changes from week 18 to weeks 30 and 42 are visualised in Fig 3 (underlying data in Table 2). The four most abundant genera *Lactobacillus*, *Bifidobacterium*, *Bacteroides* and *Prevotella* underwent significant changes in average relative abundance between week 18 and 42. *Bacteroides* and *Prevotella* significantly increased and *Bifidobacterium* and *Lactobacillus* significantly decreased with age. Those changes were also reflected at the phylum level. Bacteroidetes (the phylum that the genera *Bacteroides* and *Prevotella* belong to) increased whilst Actinobacteria (*Bifidobacterium*) and Firmicutes (*Lactobacillus*) decreased. *Megasphaera* underwent the biggest change with age, from 0.1% and 0.2% at week 18 and week 30 to 8.4% at week 42, an odds ratio (OR) of 85 (Table 2 and S6 Table).

**Table 1:** Summary of statistically significant time effects on taxonomy and functional potential of the feline faecal microbiome.

	Identified	Significant	Consistentlyincreasing	Consistently decreasing
**Genera**	605	589	585	4
**Families**	238	233		
**Ortholog groups**	7,277	3,617	2,491	1,007
**Biochemical pathways**	190	130		
**Pathway groups**	32	26		

Consistently increased/decreased denotes elements with significantly different odds in the same direction when comparing weeks 18 with 30 and weeks 18 with 42. Full statistical results are provided in S6 Table.

**Table 2:** Average percentages of most abundant genera and phyla.

		Week 18 [%]	Week 30 [%]	Week 42 [%]
**Actinobacteria**		**23.5**	17.5	11.8
	*Atopobium* ^+^	0.3	0.5	0.3
	*Bifidobacterium* ^-^	19.7	10.1	5.6
	*Coriobacterium* ^+^	1.1	2.4	1.7
	*Eggerthella* ^+^	0.4	0.7	0.6
	*Mycobacterium* ^+^	0.2	0.3	0.3
	*Olsenella* ^+^	0.4	0.7	0.5
	*Slackia* ^+^	0.2	0.4	0.3
	*Streptomyces* ^+^	0.3	0.5	0.4
**Bacteroidetes**		**10.2**	**25.1**	**34.7**
	*Alistipes* ^+^	0.1	0.5	0.6
	*Bacteroides* ^+^	5.5	11.8	15.5
	*Odoribacter* ^+^	0.1	0.3	0.6
	*Parabacteroides* ^+^	0.3	0.8	1.1
	*Prevotella* ^+^	2.9	9.3	13.8
**Firmicutes**		**52.8**	**40.7**	**36.1**
	*Acidaminococcus* ^+^	0.3	0.5	0.5
	*Bacillus* ^+^	0.5	0.7	0.7
	*Butyrivibrio* ^+^	0.5	0.8	0.7
	*Cellulosilyticum* ^+^	0.2	0.3	0.3
	*Clostridium* ^+^	4.4	6.9	6.0
	*Enterococcus* ^+^	0.2	0.5	0.3
	*Eubacterium*	4.3	5.3	4.3
	*Lactobacillus* ^-^	34.5	12.4	3.7
	*Megasphaera* ^+^	0.1	0.2	8.4
	*Oscillibacter* ^+^	0.5	0.8	0.7
	*Paenibacillus* ^+^	0.2	0.4	0.4
	*Roseburia* ^+^	2.0	2.6	2.1
	*Ruminococcus* ^+^	0.4	0.5	0.4
	*Selenomonas* ^+^	0.3	0.6	0.6
	*Streptococcus* ^+^	0.7	0.9	0.9
**Proteobacteria**		**9.3**	**10.5**	**11.4**
	*Burkholderia* ^+^	0.4	0.6	0.6
	*Desulfovibrio* ^+^	0.2	0.5	0.5
	*Escherichia* ^-^	1.0	0.1	0.5
	*Pseudomonas* ^+^	0.3	0.4	0.4
	*Shewanella* ^+^	0.2	0.3	0.3
**Spirochaetes**		**0.8**	**1.1**	**1.0**
	*Treponema* ^+^	0.3	0.5	0.5
**Total (phyla**)		**96.6**	**94.9**	**95.0**
	Total (genera)	83.2	74.1	74.3

Shown are all 34 genera with ≥ 0.3% average relative abundance at ≥ 1 time point. Phylum abundance includes all genera within the respective phyla and is not limited to the genera displayed. Superscript plus and minus symbols indicate significantly increased and decreased odds with age with *p*<0.00008. Abundance data for individual samples and results of the statistical analyses are provided in S4 and S5 Tables.

In total, 5,196,048 unique protein-coding genes were identified in the microbiomes of feline faeces (4,184,928 at week 18, 4,398,745 at week 30 and 4,503,184 at week 42). The number of unique protein-coding genes significantly increased between weeks 18 and 30 as well as weeks 18 to 42 but not between weeks 30 and 42 at *p*<10^−5^ (Fig 1C and S5 Table). No other significant associations were found at *p*<0.05. Of the significant genes, 3,486,858 (67%) had a KEGG annotation that was used to relate them to 7,277 KEGG ortholog groups (KEGG level 4; S2 Table). Similar to the genus data, a PCoA of the ortholog group relative abundance data indicated a structure mostly consistent with age (Fig 2B) and a dominant first component at 63%. Higher levels of functional annotation supported a similar structure (S3 Fig).

Ortholog groups mapped to 253 biochemical pathways (KEGG level 3) of which 190 were retained after a parsimony approach was applied (MinPath [30]; Table 3). Pathways were organized into 32 groups (KEGG level 2) and 6 supergroups (KEGG level 1). Similar to the taxonomy section, the distribution of individual ortholog groups was skewed. Only 12.2% of the ortholog groups contributed to more than 80% of the sequences, while 53.5% of ortholog groups contributed to less than 1%.

**Table 3:** Number of biochemical pathways by pathway supergroup.

ID	Name	Raw	Parsimonious	Significant	Enriched
**L1_1**	Metabolism	136	127	91	4
**L1_2**	Organismal Systems	32	11	9	0
**L1_3**	Environmental Information Processing	14	7	4	0
**L1_4**	Cellular Processes	16	7	4	2
**L1_5**	Human Diseases	36	20	8	0
**L1_6**	Genetic Information Processing	19	14	13	2
	Total	253	190	129	8

Given are the numbers of biochemical pathways (KEGG level 3) by pathway supergroup (KEGG level 1) as identified by MetaCV[29] (Raw), retained after MinPath[30] filtering (Parsimonious), with statistically significant associations with age at *p*<0.00027 (Significant) and with a significant (*p*<0.00027) enrichment in ortholog groups (KEGG level 4) that have significant age differences at *p*<8x10^-6^ (Enriched).

Functional annotations were analysed by generalized estimating equations for associations with age, gender, neutering and age when neutered (see Table 1 for a summary). Statistically significant associations with age were identified for 3,617 out of 7,277 ortholog groups, 130 out of 190 biochemical pathways and 26 out of 32 pathway groups with adjusted *p*-values of *p*<8x10^-6^, *p*<0.00027 and *p*<0.00162, respectively (S6 Table). Similar to the taxonomy section, no significant associations with gender or neutering were found at the given *p*-value thresholds. A breakdown of pathways by supergroup is provided in Table 3.

**Fig 4:**
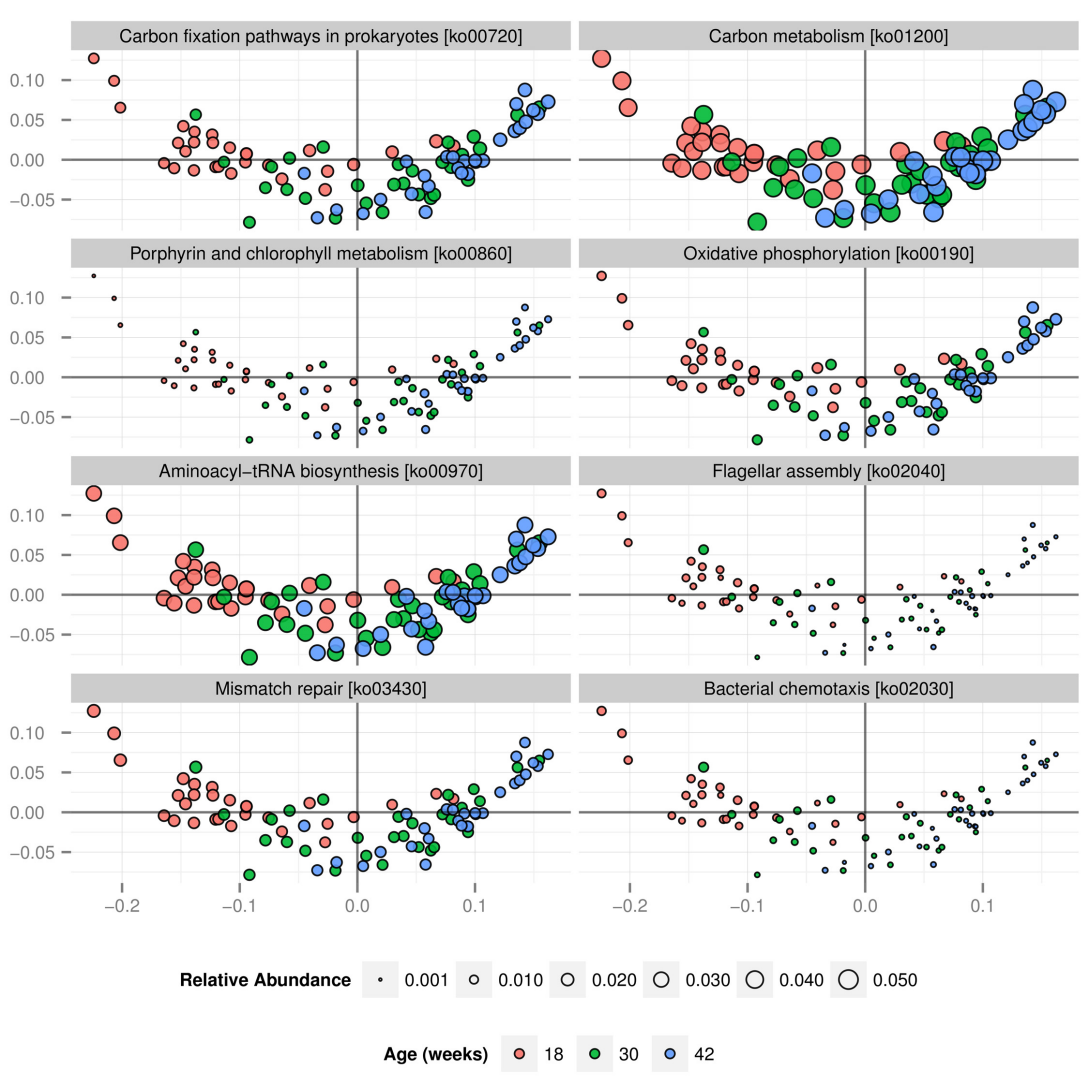
Principal coordinates analysis (PCoA) of eight biochemical pathways. Shown are eight pathways enriched in ortholog groups with significant differences with age (five increasing and three decreasing with age). Ordination was calculated based on abundance data for all ortholog groups and a Jason-Shannon divergence (JSD) measure. Data points are scaled by the relative abundance of the respective pathway in the different samples.

Ortholog groups with significant changes with age were split into groups of increasing or decreasing odds and related to KEGG pathways. A permutation test identified five and three pathways that were significantly enriched in enzymes that had been identified as significantly increased or decreased, respectively (Tables 3 and 4). Fig 4 visualises these eight pathways in a PCoA plot using the same ordination as is Fig 2B but scaling data points by the relative abundance of the respective pathway across samples. Data points were generally arranged in groups of similar size (relative abundance). This indicates that data for the eight enriched pathways was generally in agreement with data for all ortholog groups (ordination of the samples). This visualisation also helped illustrate why some samples may have been grouped with samples of other time points. For example the leftmost sample from week 30 was grouped within samples from week 18 and relative abundance of the eight pathways illustrated is more similar to other samples from week 18 that to those from week 30 (especially for chemotaxis and flagellar assembly).

**Table 4:** Biochemical pathways with over representations of ortholog groups with significant time effects.

L3 ID	L3	L2	Direction	Total	Significant
**ko00720**	Carbon fixation pathways in prokaryotes	Energy metabolism	Up	96	50
**ko01200**	Carbon metabolism	Overview	Up	240	117
**ko00860**	Porphyrin and chlorophyll metabolism	Metabolism of cofactors and vitamins	Up	87	55
**ko00190**	Oxidative phosphorylation	Energy metabolism	Up	102	65
**ko00970**	Aminoacyl-tRNA biosynthesis	Translation	Up	41	25
**ko02040**	Flagellar assembly	Cell motility	Down	41	26
**ko03430**	Mismatch repair	Replication and repair	Down	29	11
**ko02030**	Bacterial chemotaxis	Cell motility	Down	26	16

Shown are all eight biochemical pathways (KEGG level 3) that were significantly enriched (at *p*<0.00027) in ortholog groups with significant and consistent age effects (that is they contain more ortholog groups (KEGG level 4) with significant increasing or decreasing age effects than would be expected at random; see methods for details). Counts presented are the number of ortholog groups identified in feline faeces within this pathway (Total) and the number of ortholog groups with significant age effects at *p*<8x10^-6^ (Significant).

## Discussion

### No evidence for an effect of sexual development on the microbiome of growing kittens

We studied the faecal microbiome of growing kittens to identify potential associations with age, gender and sexual development (neutering and neuter age). To minimize any dietary effects, kittens were fed a commercially available diet produced during a single production run and energy intake was adjusted to maintain optimal body condition scores [31]. Under these conditions we only found statistically significant associations with age. This study did not provide evidence for significant effects of neutering, age when neutered or gender. These findings were consistent for analyses of diversity, taxonomy and functional potential.

Neutering of the cats in this study was required as part of a welfare strategy, and so sexual development was stopped prior to achieving reproductive age (average of 8.5–10 months of age [32]). However, sexual development would have started prior to 19 weeks of age, as sex hormones are detected in cat faeces, with puberty achieved by 13–14 weeks of age [33]. By 31 weeks of age, the conventional neutered cats were a similar age to others (aged seven months), previously classified as post-pubertal when neutered [32]. More specifically, metabolite profiling of the kittens used in the study described here [34], identified that plasma metabolites were significantly different between neuter groups by 26 weeks of age, indicative of an effect of sexual development on host metabolism. In this study, sexual development to 31 weeks did not have a significant effect on the faecal microbiota, however, it remains possible that late stage sexual maturation may affect GI flora.

The absence of gender differences in the faecal microbiome is consistent with other published work. To our knowledge only one study published to date reported a sexual dimorphism in the microbiomes of healthy individuals [35]. However, that study investigated Hadza hunter-gatherers who practice a gender based division of labour and it was concluded that the differences were diet induced and a consequence of snacking from different foods throughout the day.

It should also be noted that a lack of statistically significant effects of neutering, neutering age and sex does not categorically exclude that these factors may have an impact. It is possible that these effects may be small and the current study is not powered to detect such small effect sizes.

### Temporal dynamics of the feline faecal microbiome

Structural changes to the faecal microbiome were largely driven by five genera: *Lactobacillus*, *Bifidobacterium*, *Bacteroides*, *Prevotella* and *Megasphaera*. Relative abundance of *Lactobacillus* and *Bifidobacterium* consistently decreased with age while *Bacteroides* and *Prevotella* consistently increased with age. *Megasphaera* showed a different pattern, being rare at weeks 18 and 30 (0.1% and 0.2% average abundance) and the third most abundant genus at week 42 (average abundance 8.4%). The decrease in *Lactobacillus* and *Bifidobacterium* was not compensated by the increase in *Bacteroides* and *Prevotella* (cumulative abundances of 62.5%, 43.7% and 38.7% at weeks 18, 30 and 42, respectively). Instead, a large number of rare genera increased in relative abundance, which is consistent with an increase in Shannon diversity from 3.9 to 4.8 and 4.9, respectively. The relatively small increase from week 30 to 42 and the lack of statistical significance may indicate that the diversity was mostly established by week 30. This conclusion is also supported by a significant increase in the unique number of protein coding genes between week 18 and 30 and a lack of significance of the increase between week 30 and 42.

Bifidobacteria are predominant in human infant faeces [36,37] and have also been detected in feline faeces [38–40]. Studies in pigs have reported a decrease in *Lactobacillus* after weaning [41,42], often explained by changes in substrate availability and gastric pH. *Bacteroides* and *Prevotella* have gained attention in the human field, the former being associated with diets high in protein and animal fats and the latter with diets high in carbohydrates [3]. We have previously identified a similar association in cats with those fed a high protein diet having increased *Bacteroides* and those fed a moderate protein diet having increased *Prevotella* [16]. A more recent study in humans, which included infants, described a strong orthogonal gradient between *Bifidobacterium* and the *Bacteroides*/*Prevotella* trade-off [4] which is consistent with the findings of this study. In summary, the changes to *Lactobacillus*, *Bifidobacterium*, *Bacteroides* and *Prevotella* are consistent with a maturing microbiome in other species. *Megasphaera* showed an 87-fold increase in the odds of being observed when comparing week 18 and 42 representing the biggest age effect on the genus level. In a previous study *Megasphaera* also showed the largest increase in odds (OR 152) when the microbiomes of kittens fed a high and a moderate protein diet were compared [16,17]. In our reference database the genus is represented by *M*. *elsdenii* [43], a gram-negative ruminal bacterium with the ability to ferment lactate to short-chain fatty acids [44,45]. *M*. *elsdenii* has been shown to have beneficial effects in rats [46] and pigs [47]. *Megasphaera* is also used as probiotic in cattle, but it is beyond the scope of the current study to speculate on health effects in cats.

Out of the 589 genera that showed statistically significant differences with age, only four decreased in abundance while the remaining 585 increased. In addition to *Lactobacillus* and *Bifidobacterium* mentioned above, *Escherichia* and *Shigella* (average relative abundances of 0.05% and 0.02%) decreased over time. Only 16 genera had no significant associations with age. With the exception of *Eubacterium* all those genera had low average relative abundances (<0.5%). *Eubacterium* had a high average abundance of 4.7% making it the 6^th^ most abundant genus overall. Of note is the observation that 97% of the identified genera showed statistically significant differences over time. This occurred even with the strict control of the family wise error rate, by method of Sidak, with a significance threshold of *p*<0.00008. The large number identified as significant is most likely a consequence of using proportions to describe bacterial communities and does not necessarily imply biological relevance.

### A comparison to previous studies of the feline faecal microbiome

We have previously published on the faecal microbiota and microbiomes of kittens at 8, 12 and 16 weeks of age [16,17]. These studies demonstrated significant taxonomic differences between a high and a moderate protein diet while no significant time effects were detected. A comparison of the genera that were identified or even the diversity measures across studies is challenging because multiple factors are different between the studies (including but not limited to diet and age range) making it impossible to draw accurate conclusions. We therefore considered it more appropriate to compare whether similar statistically significant trends existed between studies. In this case any age effects observed in these studies can be compared. Taken together the two studies might suggest a scenario where the feline faecal microbiome is relatively stable early in life (8 to 16 weeks of age), undergoes a period of change (18 to 30 weeks) and then stabilises again (30 to 42 weeks), but diet differences might be sufficient to explain this pattern. Another explanation for the lack of significant age differences in the earlier study could be attributed to the shorter time frame (8 weeks vs. 24 weeks) and the smaller number of individuals (12 vs. 30 cats) making the earlier study less suitable for detecting significant age differences. Published temporal studies have also suggested gradual changes until a mature microbiome is reached [48]. Testing this hypothesis would require another study tracking the microbiome development of kittens fed a constant diet from weaning into adulthood.

### Antibiotics and the faecal microbiome

Other factors, such as antibiotics, may also impact on the gut microbiota. In this study all cats were provided with a sub-cutaneous injection of an antibiotic at the time of neutering, and as no significant effect of age when neutered was observed, it would indicate that any effect of this antibiotic treatment was no longer detectable 11 weeks after neutering. However, within the study, one cat, Winston, was prescribed an oral β-lactam antibiotic, ceporex, for seven days (twice daily) in response to a skin infection. This treatment occurred between one to three weeks before the 30 week faecal sample was taken. Winston had a large proportion of *Lactobacillus* (S2 Table), so much so, that he was an outlier in the hierarchical cluster analysis (S2 Fig). By week 42 Winston was within a sub-cluster. This evidence may be used to suggest that the antibiotic had an acute effect on microbiome structure. This is consistent with the response to oral antibiotics in humans, where *Lactobacillus* also increased proportionally [49]. Within 12 weeks the microbiome restored to a level similar to other cats which had not experienced oral antibiotic treatments. This is similar to reports in human studies where short-term antibiotic treatment had no long-term adverse effects on the microbiome [50].

### Differences in the microbiome’s functional potential

We sequenced approximately 5x10^9^ reads and identified 5x10^6^ unique protein coding genes belonging to 7x10^3^ KEGG ortholog groups. A statistical analysis found significant associations with age for 3,617 ortholog groups and 130 biochemical pathways. These large numbers make data interpretation in an objective manner very difficult. We considered identifying and interpreting the “strongest” functional differences to be the appropriate approach. In an earlier study [16] we have used a two-step process for this purpose that identifies biochemical pathways that are significantly enriched in ortholog gene groups that were identified as being significantly different in a preceding step. Using this definition we have successfully identified pathways of amino acid metabolism as significantly enriched between the microbiomes of two groups of kittens fed diets of different protein content [16]. In the current study we used this strategy to identify pathways enriched with ortholog groups of increased or decreased odds with time. We considered this two-step approach to be more sophisticated than using the proportions of the pathways alone as it excludes all ortholog groups without significant associations from further analysis. Among the eight pathways identified with this approach were three pathways related to energy metabolism and two to cell motility. Although our study design does not allow for an inference of causal relationships, there is value in discussing the most significant changes.

### The microbiome’s potential for energy and carbon metabolism undergoes significant changes with age

Two out of the five pathways enriched in ortholog groups with increasing odds of presence with age were related to energy metabolism: carbon metabolism and oxidative phosphorylation. Oxidative phosphorylation is a process during which ATP is generated by proton-motive force after a series of redox reactions. This finding is consistent with changes to the most abundant genus *Lactobacillus* (S2 Table), a genus of fermentative bacteria. Oxidative phosphorylation is much more efficient at generating energy than fermentation. It is therefore tempting to speculate that the microbiome becomes more efficient at harvesting energy from the diet with age. However, aerobic respiration is not advantageous in the largely anoxic gut lumen [51]. Instead we suggest that this finding is merely indicative of the relative abundance of facultative anaerobes increasing with age. This interpretation is also consistent with the observation that none of the ten species with the strongest increases in odds of being observed with age contained all enzymes for a complete oxidative phosphorylation pathway as annotated in the KEGG database (S8 Table).

Carbon metabolism represents a large collection of enzymes involved in carbon metabolism rather than a single biochemical pathway. Of the 240 ortholog groups identified in this study for this category, 117 were significantly increased in relative abundance with age. This may suggest that the microbiome is becoming capable of using a wider range of carbon sources with age. This is consistent with the bacterial community becoming more diverse, with introduction of new gene functions with new bacteria.

### Microbiome motility decreases with time

Two out of the three pathways enriched in ortholog groups with decreasing odds of presence with age were related to the KEGG category cell motility: flagellar assembly and chemotaxis. Chemotactic signalling systems allow bacteria to respond and move in response to chemical gradients [52] such as urea, bicarbonate, amino acids, or pH. Flagella are long narrow filaments on the surface of some bacteria that are important for motility to and movement within the mucus layer of the gut. Because flagella have adhesive properties to intestinal mucus [53,54] and gut epithelial cells [55], and have been reported to initiate β-defensin 2 production by host epithelial cells [56,57] they also play a role in bacterial colonization and pathogenesis. A decrease in cell motility related genes is widely consistent with significant changes to the most abundant bacterial genera with many flagellated bacteria decreasing and non-flagellated bacteria increasing in relative abundance. *Bacteroides*, *Prevotella* and *Megasphaera* do not have flagella while *Lactobacillus ruminis* (decreasing in abundance from 29% to 3%, S2 Table) is flagellated [58].

Whilst the genetic features described above could be influential for the microbes’ ability to persist, they may also be genomic features specific to bacteria that are able to thrive for different reasons. Furthermore, metagenomics data only indicate functional potential and not whether genes are actually transcribed and translated into protein. For example, motility-related genes are highly abundant in healthy human gut microbiomes [11,59], yet the levels of flagellin protein are low [60] and a study in rodents has demonstrated that the host is able to down-regulate bacterial genes related to motility [61].

## Summary

We studied the faecal microbiome of kittens at 18, 30 and 42 weeks of age and analysed for differences associated with age, gender, neutering and neutering age. Significant associations were only identified with age and this finding was consistent for across diversity measures and multiple levels of taxonomic and functional annotation. Structural and functional diversity increased from week 18 to week 30 but not between weeks 30 and 42. The earliest microbiome samples were dominated by *Lactobacillus* and *Bifidobacterium* while *Bacteroides*, *Prevotella* and *Megasphaera* were predominant genera in the later samples. The strongest changes in functional potential were in pathways related to energy metabolism and cell motility consistent with changes to the most abundant bacteria.

We conclude that the feline faecal microbiome is predominantly determined by age when diet and environment are controlled for. We suggest this finding may have implications on studies of the developing human microbiome where control over such factors is usually limited.

## Materials and Methods

### Animals and experimental design

Kittens were housed and treated in accordance with the UK Animals (Scientific Procedures) Act 1986 and the project was approved by the WALTHAM Animal Welfare and Ethical Review Body. Thirty kittens (16 male, 14 female) were recruited from 17 litters to the trial between 14 and 18 weeks of age (range 22 days). The kittens were assigned to one of four groups so that no group had more than 1 representative of a litter. The groups were based on gender (F or M) and age when neutered. Neutering was performed as part of normal veterinary practice at WALTHAM and occurred at one of two time points, one defined as early (EN), at 19 weeks of age and the other, defined as conventional (CN), at 31 weeks of age. The groups were balanced for gender across the neuter groups, consisting of 7 Female EN, 7 Female CN, 8 Male EN and 8 Male CN. All kittens received a sub-cutaneous antibiotic injection (Clamoxyl LA Inj, 15mg per kg bodyweight) as part of the neutering procedure. One kitten (Winston) required an oral β-lactam antibiotic, ceporex, for seven days (twice daily) in response to a skin infection (one to three weeks before the 30 week faecal sample was taken). One female (Duchess, EN group) was removed during the trial for non-trial related reasons and only the week 18 sample was taken. Kittens were housed in the same environmentally enriched housing in two social groups (based on gender) and cared for according to WALTHAM kitten socialisation guidelines. Kittens were individually fed to maintain an ideal body condition score (WALTHAM S.H.A.P.E. guide [31]), with weekly assessments to determine any intake changes.

### Diet

Kittens had free access to fresh water at all times throughout the study and were fed from a single batch of a nutritionally complete (National Research Council 2006) commercial dry diet (Royal Canin Kitten, 10Kg, Aimargues, France) for a minimum of 17 days before the first faecal collection.

### Fresh faecal sample collection

Samples were collected at three time points, 12 weeks apart. The oldest kittens at these sampling time points were 18, 30 and 42 weeks of age and this nomenclature is used to describe the sampling points. Exact kitten ages are specified in S9 Table. Faecal samples were collected within 15 min of defecation from all kittens and stored immediately at -80°C.

### DNA Extraction and Illumina Sequencing

Bacterial DNA was extracted using a QIAamp DNA stool mini kit (Qiagen, Valencia, CA, USA) using the repeated bead beating plus column (RBB+C) method [62]. Faecal DNA was quantified using a NanoDrop ND-1000 spectrophotometer (NanoDrop Technologies, Wilmington, DE, USA). DNA quality was assessed before Illumina sequencing using a 2100 Bioanalyzer (Agilent Technologies, Santa Clara, CA). Illumina sequencing was performed at the W. M. Keck Center for Biotechnology at the University of Illinois using an Illumina HiSeq2000 sequencer (Illumina Inc., San Diego, CA) according to manufacturer’s instructions. Briefly, shotgun genomic libraries were prepared with Illumina's Nextera DNA Sample Preparation Kit from 50ng of DNA according to the manual. The libraries were pooled in equimolar concentration into 11 pools. The pools were quantitated by qPCR with the Kapa SYBR FAST Universal (Kapa Biosystems). Each pool was sequenced on one lane for 101 cycles from each end of the fragments on a HiSeq2000 using a TruSeq SBS sequencing kit version 3. The fastq files were generated and demultiplexed with Casava1.8.2 (Illumina). Sequence reads for all 88 samples were deposited to the European Nucleotide Archive (ENA) under the project accession PRJEB9357 (sample accessions ERS723589 –ERS723676).

### Sequence data annotation

Sequence reads were trimmed using Trim Galore! version 0.3.1 with cutoff of 30 and 80 for phred score and length, respectively. Trim Galore! is a wrapper tool around Cutadapt and FastQC and all three programs are provided by Babraham Bioinformatics at http://www.bioinformatics.babraham.ac.uk/projects/. Trimmed short reads were annotated using a composition vector approach as implemented in MetaCV version 0.230 [29] against a reference database of 11,001,586 genes from 2,059 fully-sequenced genomes (supplied with MetaCV). MetaCV results files contain the following data entries for each individual short read in the FASTQ files of the respective samples: Read identifier, quality score of the annotation, GI number of the best protein match, KEGG entry identifier (if available), eggNOG identifier (if available), NCBI taxon ID and strain name. MetaCV applies a lowest common ancestor (LCA) approach on the NCBI taxonomy tree when reliable annotation down to strain level is not possible. Results were filtered by a quality score of 20 and collated into a summary table using MetaCV. Summary tables are automatically generated for the following levels of taxonomical hierarchy: superkingdom, phylum, class, order, family and genus. A summary table on the species level (which is required for the calculation of species diversity) was generated separately by parsing the results files. In terms of functional annotation, summary tables were automatically generated for KEGG [63] orthology. KEGG provides a hierarchy of ortholog groups (KEGG level 4), biochemical pathways (KEGG level 3), “pathway groups” (KEGG level 2) and “pathway supergroups” (KEGG level 1). Minpath [30], which uses a parsimony approach to pathway inference, was used to remove pathways that are likely to be erroneously inferred due to single ortholog groups being related to multiple pathways. Uniprot tables of April 2012 were used to relate genes to KEGG orthology groups and eggNOG clusters. KEGG tables of May 2011 were used to relate ortholog groups to pathways and higher level groupings. All abundance data was converted to proportions (relative abundances) for subsequent analyses.

### Richness and diversity

Diversity was estimated by calculation of Shannon indices [64] from the normalized species counts for each sample according to the formula

$$H^{\prime }=-\sum_{i=1}^{R}p_{i}\;\ln\;p_{i}$$

where R is the number of species observed and p_i_ is the proportion of the i-th species. These were then analyzed by linear mixed models with kitten as a random effect and gender, neuter date, age and their interactions were explored as categorical fixed effects. Fixed effects were tested against a cut-off of *p*<0.05 and dropped from the model if non-significant. For the final model, comparisons between groups were made by Tukey HSD tests using a family-wise 5% level. Shannon indices of individual samples and the results of the statistical analysis are provided in S3 Table.

Richness and diversity were also estimated by calculation of rarefaction curves. Strain level annotations were extracted from the MetaCV results files and random permutations were generated by using the Linux command *shuf*. To reduce noise a higher QC cutoff of 40 was applied. Sub-samples were taken at depths of 10k (thousand), 20k, 50k, 100k, 250k, 500k, 1m (million), 2m, 4m, 6m, 8m, 10 and 20m reads (unpaired) with ten replicates per depth and sample (11,440 data points in total). These were then analysed by linear mixed models with sequence depth nested in age nested in kitten as the random effects, with weighting by sequence depth variability. Neuter date, gender, age, sequence depth and their interactions were fitted as categorical fixed effects. Effects were tested against a cut-off of *p*<0.05 and dropped from the model if non-significant. In addition, the heterogeneity of variances between sequence depths was tested by likelihood ratio tests for nested random effects models. The number of species per sub-sample and the results of the statistical analysis are reported in S4 Table. Note: If annotation was not possible down to strain level, species level annotation was used for the rarefaction analysis. As a consequence the number of taxa observed may exceed the number of strains in the database (2,059).

### Principal Coordinates Analysis (PCoA)

Ordination was performed using the dudi.pco() function from R package ade4 v1.6.2 [65] with the Jensen-Shannon divergence (JSD) as a distance measure [66] calculated from the relative abundance of genera or ortholog groups (KEGG L4, Fig 2). The PCoA vectors were plotted using R package ggplot2 v1.0.0 [67]. Shape areas were weighted by the relative abundance of the respective biochemical pathway (KEGG L3) in the individual samples for Fig 4.

### Summarised visualisation of community structure

Raw genus and phylum abundance data as inferred by MetaCV [29] was converted to relative abundances using the number of annotated sequence reads as a denominator on a per-sample basis. Averages were calculated as the arithmetic means over the three different time points. The six most abundant phyla were picked for visualization. Within those phyla 34 genera had an average abundance of at least 0.3% at least at one time point and were included for visualization. Data was visualized in Circos [68] version 0.63 using Tableviewer (of 7^th^ September 2012) to create a chord diagram of phylum abundance. Peripheral bubble plots of genus abundance were generated by adding scatter plots of circular glyphs.

### Statistical analyses of abundance data

Individual datasets of relative abundances for genera, families, ortholog groups (KEGG level 4), biochemical pathways (KEGG level 3) and pathway groups (KEGG level 2) were analysed univariately by generalized estimating equations, with a binomial distribution (for proportional responses) and a logit-link, to investigate the gender and/or neuter group effects with age. Kitten was specified as a random effect to account for the lack of independence in observations over time within kittens and gender, neuter date, age and their interactions were fitted as categorical fixed effects. If effects were found to be non-significant they were dropped from the model. All effects were tested against a data set type I error rate of 5% by Sidak [69] adjustment, thus the critical *p*-values used were 0.000085 for genera, 0.000215 for families, 0.000008 for ortholog groups, 0.00027 for biochemical pathways and 0.0016 for pathway groups for 605, 238, 6427, 190 and 32 responses respectively. Prior to fitting of the univariate model sets, potential responses were filtered if the proportion of zero counts was greater than 75%. All odds ratios (OR) reported were significant at the respective adjusted *p*-value cut-off.

### Enrichment analysis of functional potential

Permutation testing was performed to identify pathways enriched in ortholog groups with significantly increasing and decreasing odds with age (pathways containing more ortholog groups with significant age differences than would be expected by chance). For this purpose the set of ortholog groups with significant age differences was subset into those with consistently increasing (odds ratios are increasing from week 18 to 30 and week 30 to 42) or decreasing odds. This resulted in 2,491 groups with consistently increased, 1,007 groups with consistently decreased odds and 119 groups with mixed odds that were excluded from these analyses. For the permutation testing, the number of ortholog groups in each KEGG pathway and the subset with significant age changes was calculated. One thousand random subsets of 2,491 and 1,007 ortholog groups were then taken (to represent random significant ortholog groups) and the number found in each pathway calculated. The probability of a pathway containing more significant ortholog groups than would be expected by chance was calculated as the percentage of subsets where the random number in each pathway was greater or equal to the number of significant ortholog groups in each pathway. A Sidak [69] adjusted critical *p*-value of 0.00027 was used to maintain a 5% type 1 error rate for the 190 pathways tested.

## Supporting Information

S1 Fig Principal coordinates analyses for six levels of taxonomy. (PDF)

S2 Fig Hierarchical cluster analysis at genus level. (PDF)

S3 Fig Principal coordinates analyses for four levels of KEGG functional annotation. (PDF)

S1 Table Read count summary. (XLSX)

S2 Table Taxonomic and functional annotation and abundance data. (XLSX)

S3 Table Shannon diversity data and statistics. (XLSX)

S4 Table Rarefaction data and statistics. (XLSX)

S5 Table Gene richness data and statistics. (XLSX)

S6 Table Statistical analysis of taxa and functions. (XLSX)

S7 Table Pathway enrichment analysis. (XLSX)

S8 Table Ortholog groups in oxidative phosphorylation for the ten species with the biggest increase in odds of being observed. (XLSX)

S9 Table Kitten ages when faecal samples were collected. (XLSX)
