# Supplementary figures and images for: A Longitudinal Study of the Feline Faecal Microbiome Identifies Changes into Early Adulthood Irrespective of Sexual Development
Source: PLoS One. 2015 Dec 14;10(12):e0144881. doi: 10.1371/journal.pone.0144881 (PMC4682054; doi:10.1371/journal.pone.0144881)

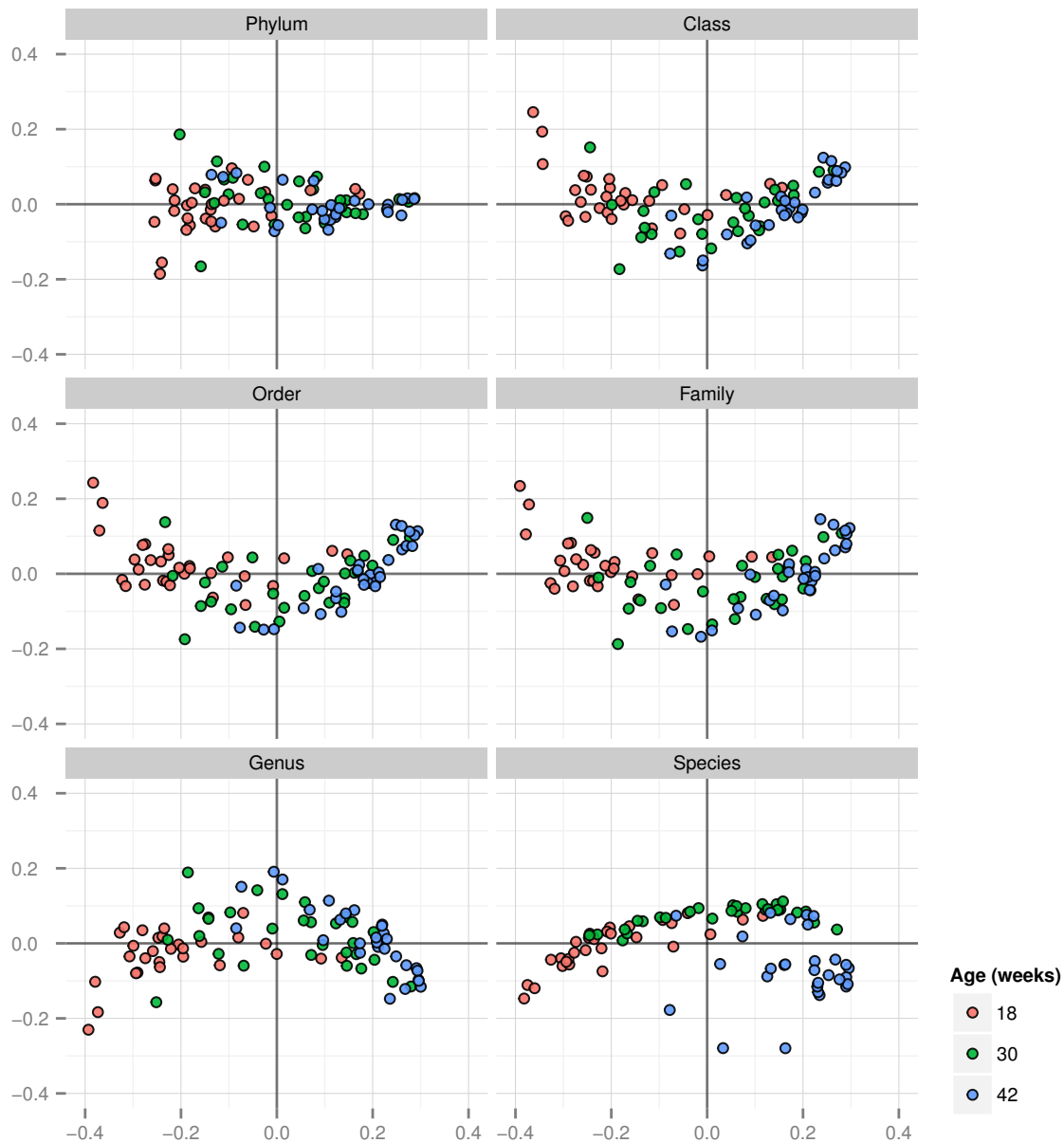

Supplement: S1 Fig — (PDF) [file pone.0144881.s001.pdf]

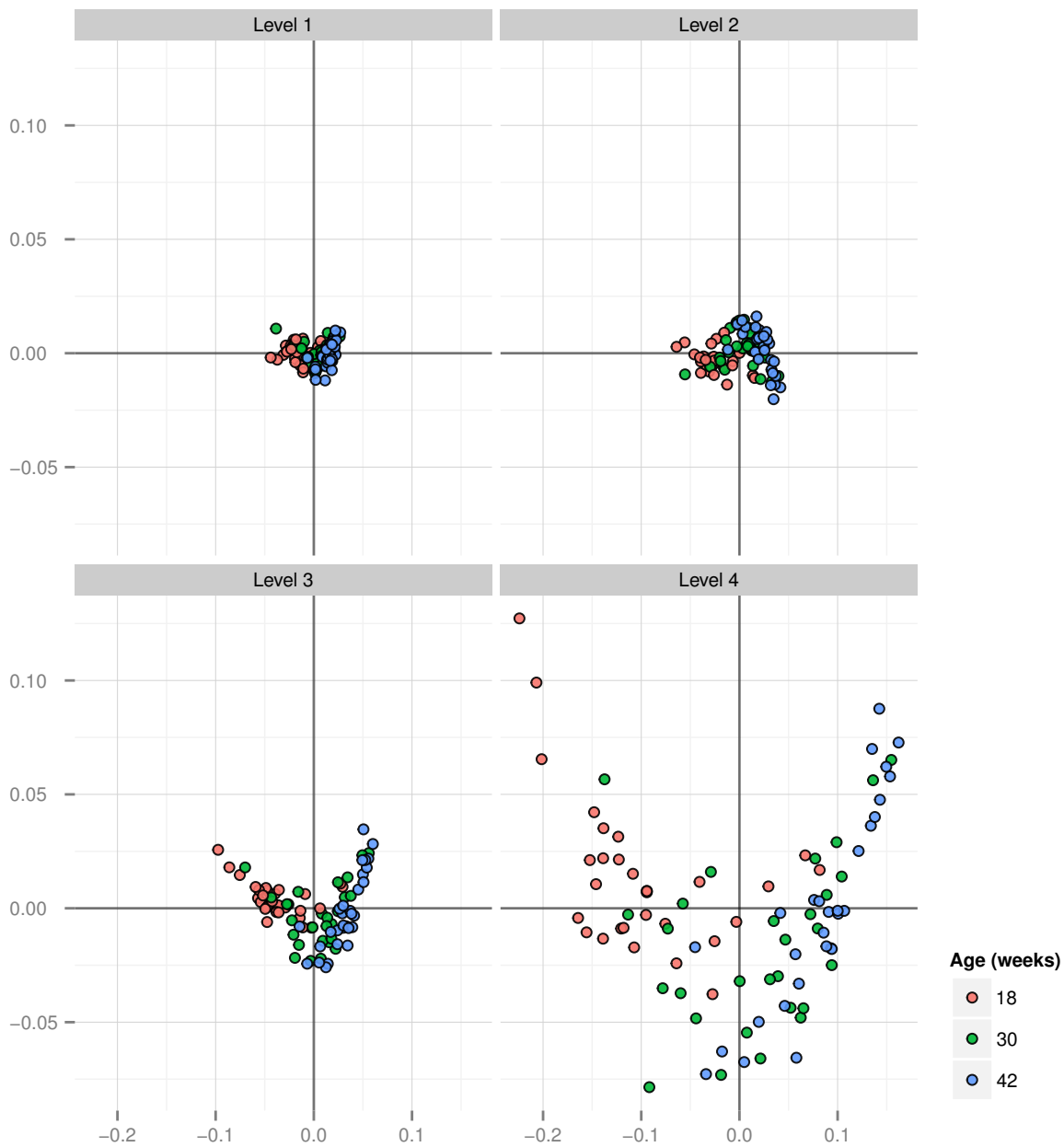

Supplement: S3 Fig — (PDF) [file pone.0144881.s003.pdf]
