# Supplementary material for: A Longitudinal Study of the Feline Faecal Microbiome Identifies Changes into Early Adulthood Irrespective of Sexual Development
Source: PLoS One. 2015 Dec 14;10(12):e0144881. doi: 10.1371/journal.pone.0144881 (PMC4682054; doi:10.1371/journal.pone.0144881)
Supplement: S2 Fig — (PDF) [file pone.0144881.s002.pdf]

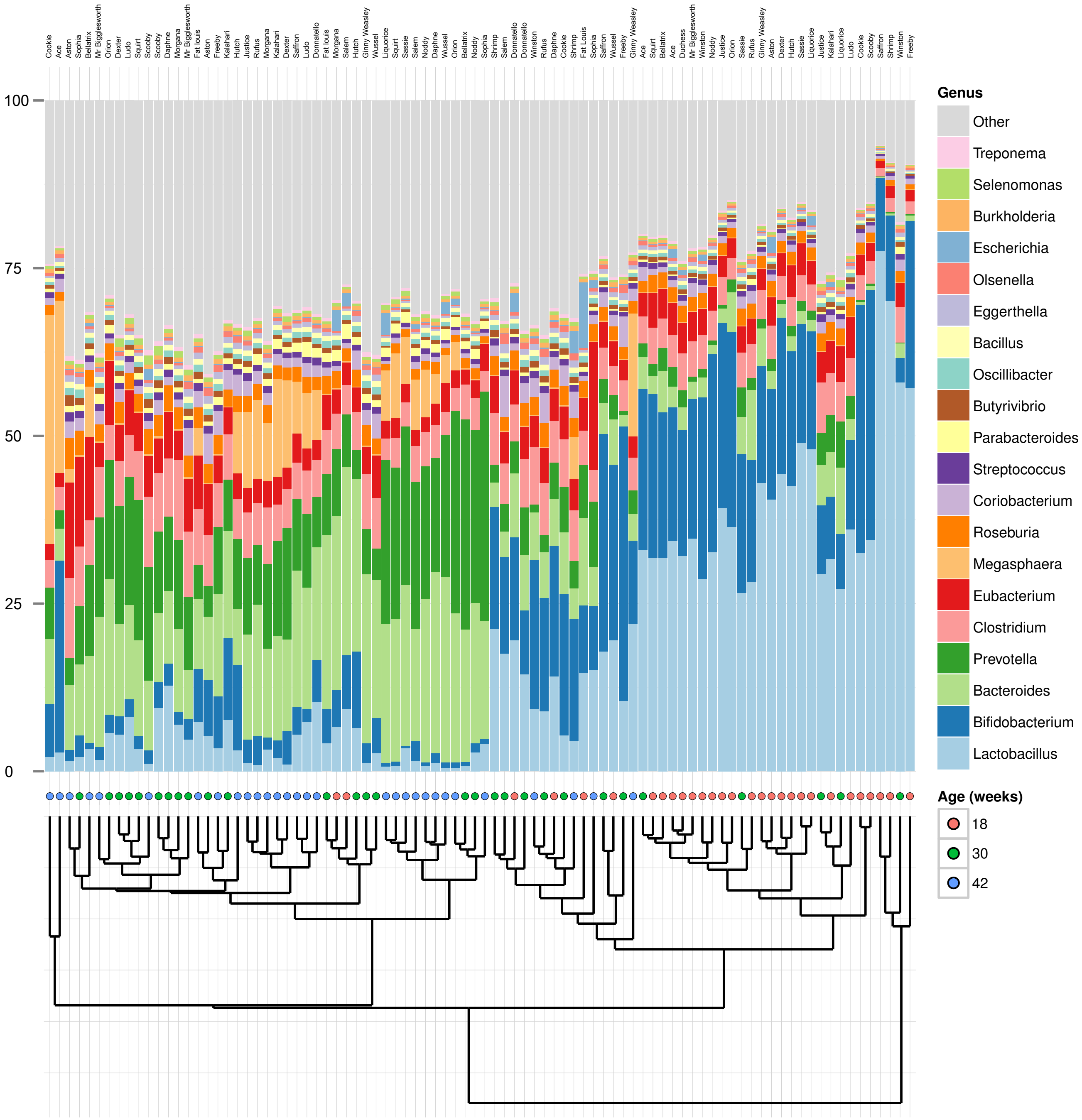

Cookie
Ace
Aston
Sophia
Bellatrix
Mr Bigglesworth
Orion
Dexter
Ludo
Squirt
Scooby
Scooby
Daphne
Morgana
Mr Bigglesworth
Fat louis
Aston
Freeby
Kalahari
Hutch
Justice
Rufus
Morgana
Kalahari
Dexter
Saffron
Ludo
Donnatello
Fat louis
Morgana
Salem
Hutch
Ginny Weasley
Wussel
Wussel
Liquorice
Squirt
Sassie
Salem
Noddy
Daphne
Wussel
Orion
Bellatrix
Noddy
Sophia
Shrimp
Salem
Donnatello
Donnatello
Winston
Rufus
Daphne
Cookie
Shrimp
Fat Louis
Sophia
Saffron
Wussel
Freeby
Ginny Weasley
Ace
Squirt
Bellatrix
Ace
Duchess
Mr Bigglesworth
Winston
Noddy
Justice
Orion
Sassie
Rufus
Ginny Weasley
Aston
Dexter
Hutch
Sassie
Liquorice
Justice
Kalahari
Liquorice
Ludo
Cookie
Scooby
Saffron
Shrimp
Winston
Freeby
100
75
50
25
0
Genus
Other
Treponema
Selenomonas
Burkholderia
Escherichia
Olsenella
Eggerthella
Bacillus
Oscillibacter
Butyrivibrio
Parabacteroides
Streptococcus
Coriobacterium
Roseburia
Megasphaera
Eubacterium
Clostridium
Prevotella
Bacteroides
Bifidobacterium
Lactobacillus
Age (weeks)
18
30
42
